# 2,3-Butanediol synthesis from glucose supplies NADH for elimination of toxic acetate produced during overflow metabolism

**DOI:** 10.1038/s41421-021-00273-2

**Published:** 2021-06-08

**Authors:** Wensi Meng, Lijie Zhang, Menghao Cao, Yongjia Zhang, Yipeng Zhang, Ping Li, Zhaoqi Kang, Shiting Guo, Ping Xu, Cuiqing Ma, Chao Gao

**Affiliations:** 1 State Key Laboratory of Microbial Technology, Shandong University, Qingdao, Shandong China; 2 Key Laboratory of Industrial Biotechnology of Ministry of Education, State Key Laboratory of Food Science & Technology, School of Biotechnology, Jiangnan University, Wuxi, Jiangsu China; 3 State Key Laboratory of Microbial Metabolism, Joint International Research Laboratory of Metabolic & Developmental Sciences, and School of Life Sciences & Biotechnology, Shanghai Jiao Tong University, Shanghai, China

**Keywords:** Cell biology, Molecular biology

## Abstract

Overflow metabolism-caused acetate accumulation is a major problem that restricts industrial applications of various bacteria. 2,3-Butanediol (2,3-BD) synthesis in microorganisms is an ancient metabolic process with unidentified functions. We demonstrate here that acetate increases and then decreases during the growth of a bacterium *Enterobacter cloacae* subsp. *dissolvens* SDM. Both bifunctional acetaldehyde/ethanol dehydrogenase AdhE-catalyzed ethanol production and acetate-induced 2,3-BD biosynthesis are indispensable for the elimination of acetate generated during overflow metabolism. 2,3-BD biosynthesis from glucose supplies NADH required for acetate elimination via AdhE-catalyzed ethanol production. The coupling strategy involving 2,3-BD biosynthesis and ethanol production is widely distributed in bacteria and is important for toxic acetate elimination. Finally, we realized the co-production of ethanol and acetoin from chitin, the second most abundant natural biopolymer whose catabolism involves inevitable acetate production through the coupling acetate elimination strategy. The synthesis of a non-toxic chemical such as 2,3-BD may be viewed as a unique overflow metabolism with desirable metabolic functions.

## Introduction

Under aerobic conditions, fast-growing organisms use fermentation instead of respiration for energy generation and excrete large quantities of acetate^1^. Recently, acetate overflow in *Escherichia coli* (*E. coli*) was identified as a result of proteome allocation to balance the conflicting proteomic demands of biomass synthesis and energy generation^2^. Acetate accumulation inhibits microbial growth, and acetate overflow is recognized as one of the major problems limiting the industrial applications of various bacterial strains^3–6^. Biotechnologists have attempted for more than 20 years to avoid overflow-caused acetate accumulation, but have achieved little success^7^.

2,3-Butanediol (2,3-BD) is a bacterial metabolite produced from pyruvate via several intermediates including α-acetolactate, acetoin (AC), and diacetyl^8,9^. The ability to generate 2,3-BD is widely distributed in bacteria, and the Voges–Proskauer reaction, which is related to intermediates of 2,3-BD production, is a fundamental test for bacterial classification^10^. 2,3-BD biosynthesis from pyruvate involves condensation of pyruvate to AC, and the reduction of AC to 2,3-BD, a step that requires an extra NADH. Thus, the production of 2,3-BD was proposed to prevent acidification by changing an acidic molecule to a neutral compound^10^ and to consume excess reducing power^11^. However, 2,3-BD biosynthesis from glucose does not involve proton consumption and produces an extra NADH, which is contrary to the above theory. Until now, the physiological function of 2,3-BD biosynthesis in bacteria has not been clarified^12^.

In this study, a naturally existing strategy for toxic acetate elimination involving 2,3-BD biosynthesis and ethanol production was identified in *Enterobacter cloacae* subsp. *dissolvens* SDM (*E. cloacae* SDM). 2,3-BD biosynthesis from glucose was confirmed to supply the reducing power required for ethanol generation and acetate elimination. Based on the identified mechanism for acetate elimination, *E. cloacae* SDM was metabolically engineered to transform acetate produced during the N-acetylglucosamine (GlcNAc, the monomer of chitin) metabolism into ethanol. Co-production of ethanol and AC from chitin, the second most abundant natural biopolymer after cellulose, was realized.

## Results

### Acetate accumulates and then vanishes during the growth of *E. cloacae* SDM

**Fig. 1:**
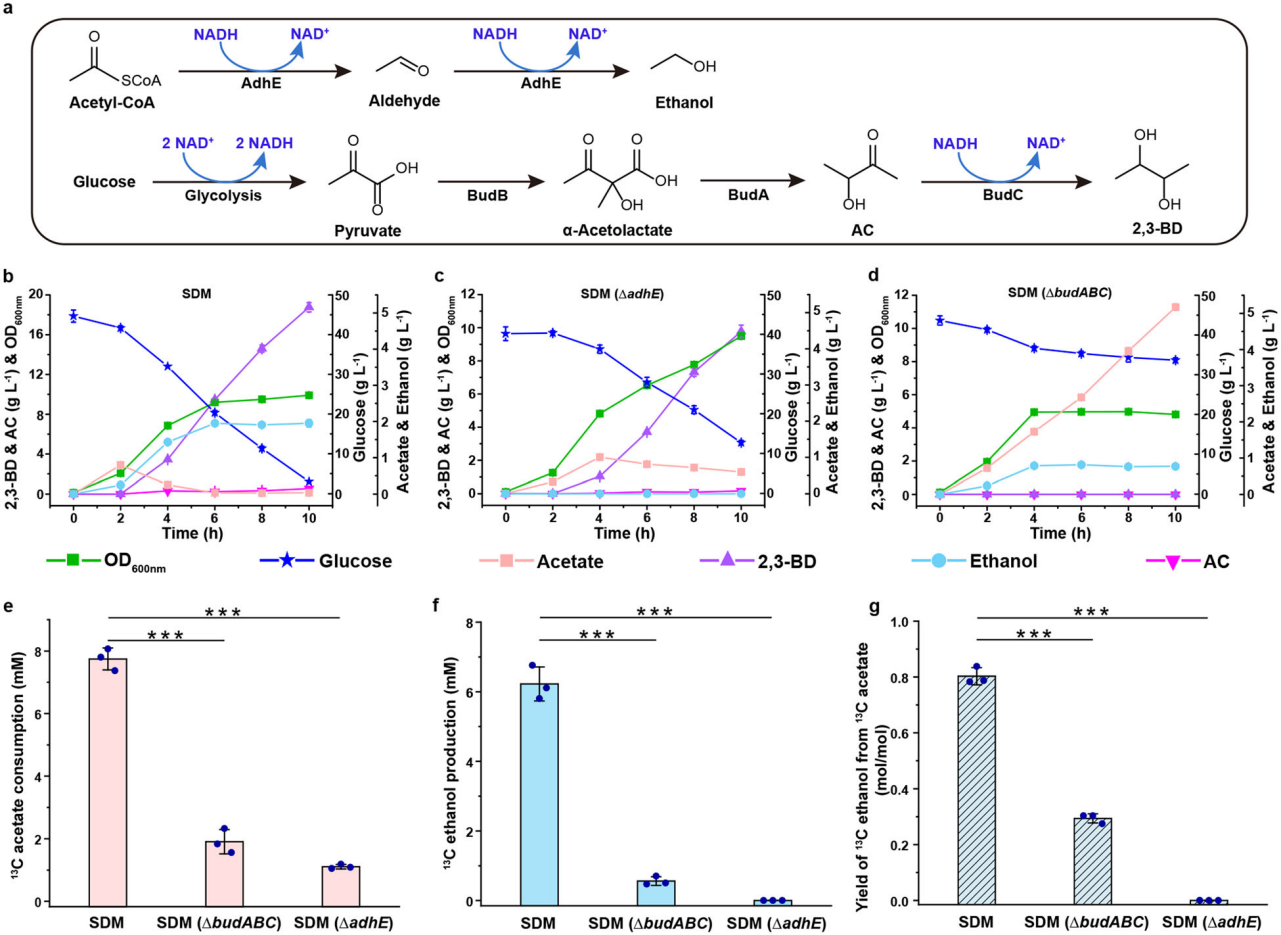
2,3-BD biosynthesis and ethanol production participate in the elimination of acetate produced by overflow metabolism. **a** Reactions related to ethanol production and 2,3-BD biosynthesis in *E. cloacae* SDM. AdhE, bifunctional acetaldehyde/ethanol dehydrogenase; BudB, α-acetolactate synthase; BudA, α-acetolactate decarboxylase; BudC, 2,3-butanediol dehydrogenase; AC, acetoin; 2,3-BD, 2,3-butanediol. **b** Growth of *E. cloacae* SDM using glucose as carbon source. **c** Growth of *E. cloacae* SDM (Δ*adhE*) using glucose as carbon source. **d** Growth of *E. cloacae* SDM (Δ*budABC*) using glucose as carbon source. **e** Consumption of exogenous added ^13^C acetate by *E. cloacae* SDM and its derivatives. **f** Production of ^13^C ethanol by *E. cloacae* SDM and its derivatives. **g** Yield of ^13^C ethanol from ^13^C acetate by *E. cloacae* SDM and its derivatives. ^13^C sodium acetate at a concentration about 10 mM was added into the culture system of *E. cloacae* SDM, *E. cloacae* SDM (Δ*adhE*), and *E. cloacae* SDM (Δ*budABC*) at 2 h. After 4 h of culture, the concentrations of ^13^C ethanol and ^13^C acetate were assayed. Experiments were carried out under aerobic conditions. Data shown are means ± SD (*n* = 3 independent experiments). ***P < 0.001 in two-tailed Student’s *t*-test.

*E. cloacae*, a Voges–Proskauer-positive strain with the ability to produce 2,3-BD (Fig. 1a), is widely distributed in gut and rhizosphere soil^13^. 2,3-BD released by *E. cloacae* was reported to promote the growth of *Arabidopsis thaliana*^14,15^. In this study, *E. cloacae* SDM was firstly aerobically cultured with glucose as the carbon source. Similar to *E. coli*, *E. cloacae* SDM underwent acetate overflow metabolism, and acetate accumulated at the beginning of its growth (2 h) (Fig. 1b). Metabolic flux analysis indicated that 55% of utilized glucose was metabolized to acetate by *E. cloacae* SDM (Supplementary Fig. S1a). Thereafter, acetate vanished (2−6 h) and carbon was redirected into 2,3-BD (73%) and ethanol (15%) (Fig. 1b; Supplementary Fig. S1b). To further identify the catabolic fate of acetate, ^13^C-labeled sodium acetate (~10 mM) was added into the culture system of *E. cloacae* SDM at 2 h and the variation of ^13^C-labeled acetate (^13^C_1_-CH_2_COOH) was detected. Our results showed that exogenous ^13^C-labeled acetate also vanished, and most of the ^13^C-labeled acetate was reduced to ^13^C-labeled ethanol by *E. cloacae* SDM (Fig. 1e–g; Supplementary Table S1).

### Acetate elimination requires 2,3-BD and ethanol production

Bifunctional acetaldehyde/ethanol dehydrogenase (AdhE) can catalyze the reduction of acetyl-CoA to produce ethanol^16^ (Fig. 1a). As shown in Fig. 1c, *E. cloacae* SDM (Δ*adhE*) lost the ability to produce ethanol and eliminate acetate, and more carbon flux was channeled to acetate (Supplementary Fig. S1c, d). A 2,3-BD biosynthesis gene cluster *budABC*, encoding α-acetolactate decarboxylase (BudA), α-acetolactate synthase (BudB), and *meso*-2,3-BD dehydrogenase (BudC), was annotated in *E. cloacae* SDM^8^ (Fig. 1a). As shown in Fig. 1d, *E. cloacae* SDM (Δ*budABC*) lost the ability to produce 2,3-BD. Interestingly, the production of ethanol also decreased, and acetate became the major metabolite of *E. cloacae* SDM (Δ*budABC*) (Fig. 1d; Supplementary Fig. S1e, f). Elimination of exogenously added ^13^C acetate by these two mutant strains was also assayed. *E. cloacae* SDM (Δ*budABC*) and *E. cloacae* SDM (Δ*adhE*) could not metabolize most of the ^13^C-labeled acetate to ^13^C-labeled ethanol as *E. cloacae* SDM (Fig. 1e–g), further supporting that 2,3-BD biosynthesis and ethanol production participate in the elimination of acetate.

### 2,3-BD synthesis is induced for acetate elimination

**Fig. 2:**
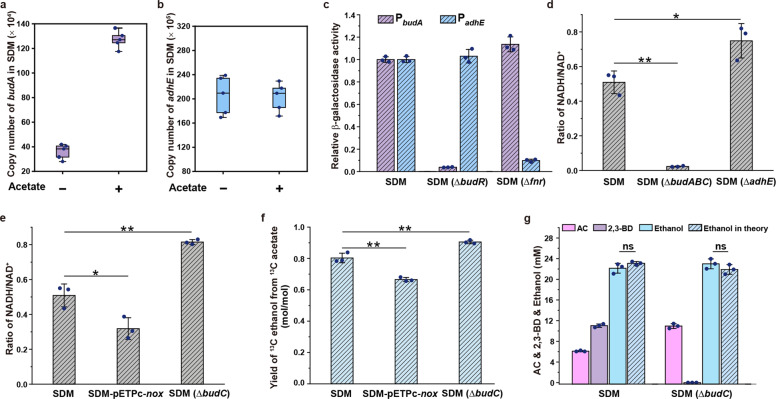
Mechanism of 2,3-BD biosynthesis supports acetate elimination. **a** Copy numbers of *budA* in *E. cloacae* SDM during growth in glucose and glucose supplemented with 40 mM sodium acetate at mid-log stage (*n* = 5). **b** Copy numbers of *adhE* in *E. cloacae* SDM during growth in glucose and glucose supplemented with 40 mM sodium acetate at mid-log stage (*n* = 5). **c** Relative β-galactosidase activity in *E. cloacae* SDM, *E. cloacae* SDM (Δ*budR*) and *E. cloacae* SDM (Δ*fnr*) containing pME6522-P_*budA*_ or pME6522-P_*adhE*_ at mid-log stage. The β-galactosidase activity in *E. cloacae* SDM is assumed to be 1. **d** Ratio of NADH/NAD^+^ in *E. cloacae* SDM, *E. cloacae* SDM (Δ*budABC*), and *E. cloacae* SDM (Δ*adhE*) at mid-log phase. **e** Ratio of NADH/NAD^+^ in *E. cloacae* SDM, *E. cloacae* SDM-pETPc-*nox*, and *E. cloacae* SDM (Δ*budC*). **f** Yield of ^13^C ethanol from ^13^C acetate in *E. cloacae* SDM, *E. cloacae* SDM-pETPc-*nox* and *E. cloacae* SDM (Δ*budC*). ^13^C sodium acetate at a concentration of about 10 mM was added into the culture system of these strains at 2 h. After 4 h of culture, the concentrations of ^13^C ethanol and ^13^C acetate were assayed. **g** Concentration of AC, 2,3-BD, ethanol, and the theoretical concentration of ethanol in *E. cloacae* SDM and *E. cloacae* SDM (Δ*budC*) when cultured under anaerobic conditions. Experiments in **a**−**f** were carried out under aerobic conditions. Data shown are means ± SD. (*n* = 3 independent experiments). *P < 0.05 in two-tailed Student’s *t*-test; **P < 0.01 in two-tailed Student’s *t*-test; ns, no significant difference (*P* ≥ 0.05 in two-tailed Student’s *t*-test).

The expression of *budA* and *adhE* during the growth of *E. cloacae* SDM was assayed by quantitative real-time PCR (qPCR). Expression of *adhE* was initiated at the beginning of growth (2 h), and *budA* was induced at 4 h and then repressed when acetate was consumed (6−10 h) (Supplementary Fig. S2a, b). When acetate was added to the medium, the expression of *budA*, but not *adhE*, was enhanced (Fig. 2a, b; Supplementary Fig. S2c, d).

In *Bacillus subtilis*, the expression of the *alsSD* operon for AC (precursor of 2,3-BD) formation was regulated by a LysR-type transcriptional regulator AlsR^17–19^. BudR, a homolog of AlsR in *B. subtilis*, was annotated upstream of BudABC in the genome of *E. cloacae* SDM. Deletion of *budR* decreased the expression of *budA*, and both 2,3-BD production and acetate elimination were blocked in *E. cloacae* SDM (Δ*budR*) (Supplementary Fig. S2e−g). However, there was no distinction in the expression of *adhE* between *E. cloacae* SDM (Δ*budR*) and the wild-type strain (Fig. 2c). The expression of *adhE* was controlled by FNR, a global regulator in *E. coli*^20^. Mutation of FNR in *E. cloacae* SDM sharply reduced the expression of *adhE* but had no influence on the expression of *budA* (Fig. 2c). Thus, although both 2,3-BD biosynthesis and ethanol production are indispensable for acetate elimination in *E. cloacae* SDM, the expression of each of these two pathways is likely to be independent of the other, and only 2,3-BD synthesis was specially induced by acetate.

### 2,3-BD synthesis from glucose provides NADH for acetate elimination

2,3-BD synthesis is indispensable for acetate elimination, which involves the production of AdhE-catalyzed ethanol from acetyl-CoA. Independent regulation of *budABC* and *adhE* supports that the role of 2,3-BD synthesis in acetate elimination may provide a particular component required for the AdhE-catalyzed reaction. Since AdhE catalyzes NADH-mediated conversion of acetyl-CoA to ethanol, effects of 2,3-BD synthesis and ethanol production on the NADH/NAD^+^ ratio were assayed. As shown in Fig. 2d, the NADH/NAD^+^ ratio in *E. cloacae* SDM (Δ*budABC*) decreased, while it increased in *E. cloacae* SDM (Δ*adhE*). Thus, both synthesis of 2,3-BD and the production of ethanol influenced the intracellular redox state. 2,3-BD synthesis provided NADH while ethanol production consumed NADH.

The above phenomenon led us to hypothesize that 2,3-BD synthesis is able to supply reducing power for AdhE-catalyzed acetate elimination. Further experiments involving redox state perturbation under aerobic conditions support our hypothesis. Decrease in the NADH/NAD^+^ ratio through the expression of water-forming NADH oxidase (NOX) from *Lactobacillus brevis* CICC 6004^9^ or using gluconic acid (an oxidation product of glucose) as a substrate reduced the efficiency of acetate elimination (Fig. 2e, f; Supplementary Fig. S2h−j). Glycolysis of one mole of glucose generates two moles of NADH and two moles of pyruvate. One mole of 2,3-BD synthesis consumes two moles of pyruvate and one mole of NADH. From the perspective of overall glucose metabolism, one mole of 2,3-BD synthesis may supply one mole of NADH. As shown in Fig. 2e, f, increasing the NADH/NAD^+^ ratio by blocking BudC, which catalyzes the NADH-requiring reduction of AC to produce 2,3-BD, also improved the efficiency of acetate elimination. This result indicates that the partial pathway ending at AC is stronger than the 2,3-BD synthesis for NADH generation.

NADH is mainly generated by glycolysis and oxidized by fermentative metabolism under anaerobic conditions^21^. Synthesis of pyruvate dehydrogenase would be repressed in the absence of oxygen^22^. Formate-lyase (PFL) becomes the main source of acetyl-CoA under anaerobic condition^23^. Formate dehydrogenase (FDH) catalyzed dehydrogenation of formate might generate extra NADH and influence the redox balance. However, there is no protein with a similarity higher than 30% to FDH of *Candida boidinii* in the genome of *E. cloacae* SDM, and no FDH activity was detected in the crude extract of *E. cloacae* SDM (Supplementary Fig. S3). Anaerobic ATP generation through glycolysis, PFL, and AdhE can convert 1 mol of glucose to 2 mol ethanol with an excess demand of 2 mol NADH. The production of 1 mol of AC from glucose could produce 2 mol of excess NADH, which means that the biosynthesis of one AC molecule supported the production of two ethanol molecules. Likewise, the production of 1 mol 2,3-BD could produce 1 mole of excess NADH, and therefore, biosynthesis of one 2,3-BD molecule supported the production of one ethanol molecule (Supplementary Fig. S2k−m). As shown in Fig. 2g, the product proportions of *E. cloacae* SDM and *E. cloacae* SDM (Δ*budC*) under anaerobic conditions coincide with the theoretical predictions, indicating that 2,3-BD synthesis participates in acetate elimination by providing excess NADH for the reactions catalyzed by AdhE.

### Acetate elimination is widely distributed in bacteria

**Fig. 3:**
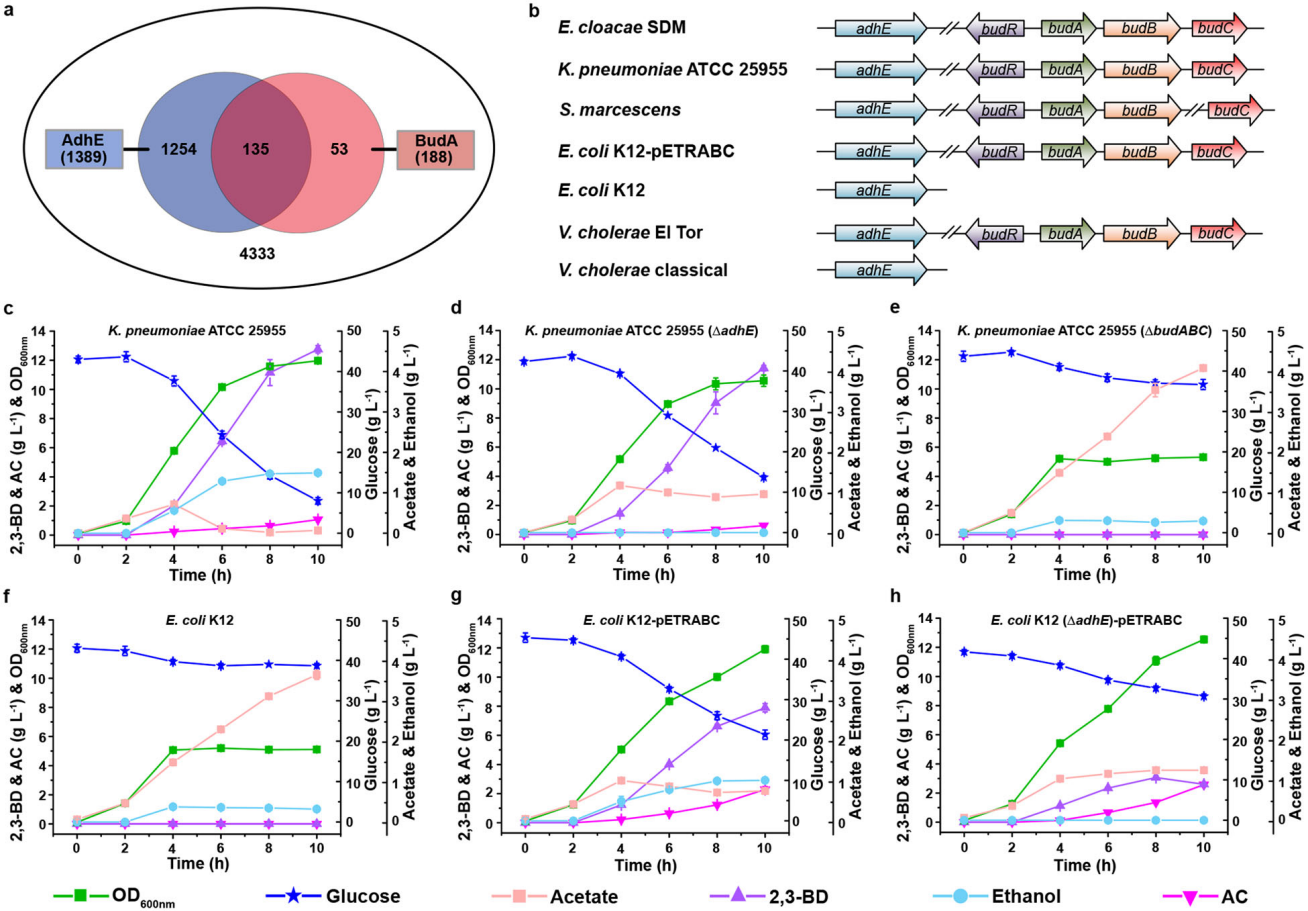
Distribution and function of BudA and AdhE in bacteria. **a** The Venn diagram illustrates the occurrence and overlap of BudA and AdhE through genome context analysis. A total number of 4333 genomes were obtained by NCBI Blast online with the restriction of maximum identity level higher than 50%, query coverage of more than 90%, and E value lower than e^−30^. **b** Organizations of 2,3-BD biosynthesis operon and *adhE* gene in different bacteria. **c** Growth of *K. pneumoniae* ATCC 25955 using glucose as carbon source. **d** Growth of *K. pneumoniae* ATCC 25955 (Δ*adhE*) using glucose as carbon source. **e** Growth of *K. pneumoniae* ATCC 25955 (Δ*budABC*) using glucose as carbon source. **f** Growth of *E. coli* K12 using glucose as carbon source. **g** Growth of *E. coli* K12-pETRABC using glucose as carbon source. **h** Growth of *E. coli* K12 (Δ*adhE*)-pETRABC using glucose as carbon source. Experiments were carried out under aerobic conditions. Data shown are means ± SD (*n* = 3 independent experiments).

The distribution of BudA and AdhE in bacteria was studied by using the Protein BLAST program in the sequenced bacterial genomes from GenBank (updated December 3, 2018). Homologs of BudA are found in the genomes of 188 bacterial species, and homologs of AdhE are found in the genomes of 1389 bacterial species (Fig. 3a; Supplementary Table S2). Among the 4333 completely sequenced bacteria, 135 species of bacteria, including *E. cloacae*, *K. pneumoniae*, and *S. marcescens*, have homologs of both BudA and AdhE (Fig. 3b).

*K. pneumoniae* ATCC 25955 was cultured aerobically in M9 medium supplemented with glucose as the carbon source. As shown in Fig. 3c, acetate accumulated at the first stage (0−4 h) and subsequently vanished (4−6 h) during the growth of *K. pneumoniae* ATCC 25955. *K. pneumoniae* ATCC 25955 (Δ*adhE*) lost the ability to produce ethanol and eliminate acetate (Fig. 3d). Additionally, *K. pneumoniae* ATCC 25955 (Δ*budABC*) lost the ability to eliminate acetate, and the growth of the strain was inhibited by the accumulated acetate (Fig. 3e). These data demonstrate that 2,3-BD synthesis and ethanol production participate in the acetate elimination process of *K. pneumoniae* ATCC 25955.

*E. coli* K12 possesses only AdhE and could not produce 2,3-BD (Fig. 3b). As expected, it accumulated acetate during aerobic growth (Fig. 3f). When *budABC* from *E. cloacae* SDM was overexpressed, *E. coli* K12-pETRABC acquired the capability to produce 2,3-BD and eliminate the acetate produced by overflow (Fig. 3g). However, when *adhE* was knocked out, continuous accumulation of acetate during the growth of *E. coli* K12 (Δ*adhE*)-pETRABC re-appeared (Fig. 3h). Therefore, the function of 2,3-BD synthesis to support acetate elimination can only be transferred into bacteria containing AdhE.

The genes *budABC* and *adhE* coexist in various bacterial species such as *K. pneumonia*, *Vibrio cholera*, and *Serratia marcescens*. Deletion of *adhE* or *budABC* resulted in higher acetate accumulation and decreased glucose utilization in both *E. cloacae* and *K. pneumonia*. Medium acidification and decreased survival due to acetate accumulation were also observed after deleting *budABC* in *E. cloacae* SDM (Supplementary Tables S3, S4). *V. cholerae* O1 has two biotypes, classical and El Tor^24^. The biotype El Tor, which possesses *budABC*, accumulated 2,3-BD without acetate accumulation, whereas the classical biotype, which lacks *budABC*, accumulated high concentrations of acetate. In addition, the 2,3-BD synthesis-positive biotype also showed higher survival rates, probably due to the absence of toxic acetate accumulation in the culture broth^25^. A similar phenotype was also observed for *S. marcescens*: this species showed a higher survival rate in the presence of *budABC*^26^. Thus, we can speculate that acetate elimination involving 2,3-BD synthesis and ethanol production is likely to be widely used in bacteria to contribute to rapid usage of carbon sources and cell survival.

### Elimination of acetate overflow requires the participation of Pta-AckA

**Fig. 4:**
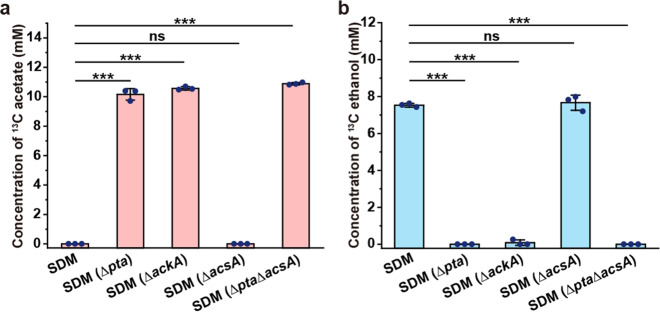
Pta-AckA rather than AcsA is essential in the biotransformation of acetate to acetyl-CoA during acetate elimination. **a** Concentrations of ^13^C-labeled acetate in *E. cloacae* SDM and its derivatives. **b** Concentrations of ^13^C-labeled ethanol in *E. cloacae* SDM and its derivatives. ^13^C sodium acetate at a concentration of about 10 mM was added into the culture system of strains at 2 h. After 4 h of culture, the concentrations of ^13^C ethanol and ^13^C acetate were assayed. Experiments were carried out under aerobic conditions. Data shown are means ± SD (*n* = 3 independent experiments). ***P < 0.001 in two-tailed Student’s *t*-test; ns, no significant difference (*P* ≥ 0.05 in two-tailed Student’s *t*-test).

Besides acetyl-CoA reduction to produce ethanol, activation of acetate to acetyl-CoA is also needed for acetate elimination^27^. Phosphotransacetylase (Pta), acetate kinase (AckA), and acetyl-CoA synthetase (AcsA) were reported to participate in acetyl-CoA production^28–30^. Assays using *E. cloacae* SDM with the knockout of AckA, Pta, and AcsA indicated that activation of acetate to acetyl-CoA is mainly executed by AckA and Pta, two enzymes expressed during acetate overflow (Fig. 4). As for the energy required for the AckA and Pta-catalyzed reaction, metabolic processes such as oxidative phosphorylation, pyruvate, 2,3-BD, and lactate synthesis could all produce ATP. However, only 2,3-BD synthesis can supply both ATP and NADH for acetate elimination with lower proteomic demands and without toxic acidic molecule production (Supplementary Fig. S4a−d). The decreased ATP concentration resulting from the mutation of *budABC* in *E. cloacae* SDM can partially support the speculation that 2,3-BD synthesis also supplies energy for the activation of acetate (Supplementary Fig. S4e). However, other experiments that could perturb ATP concentration without influencing the redox state are needed to identify the specific contribution of 2,3-BD synthesis in the energy requirements of acetate elimination.

### Acetate generated from biomass chitin can be transformed into ethanol based on elimination mechanism

**Fig. 5:**
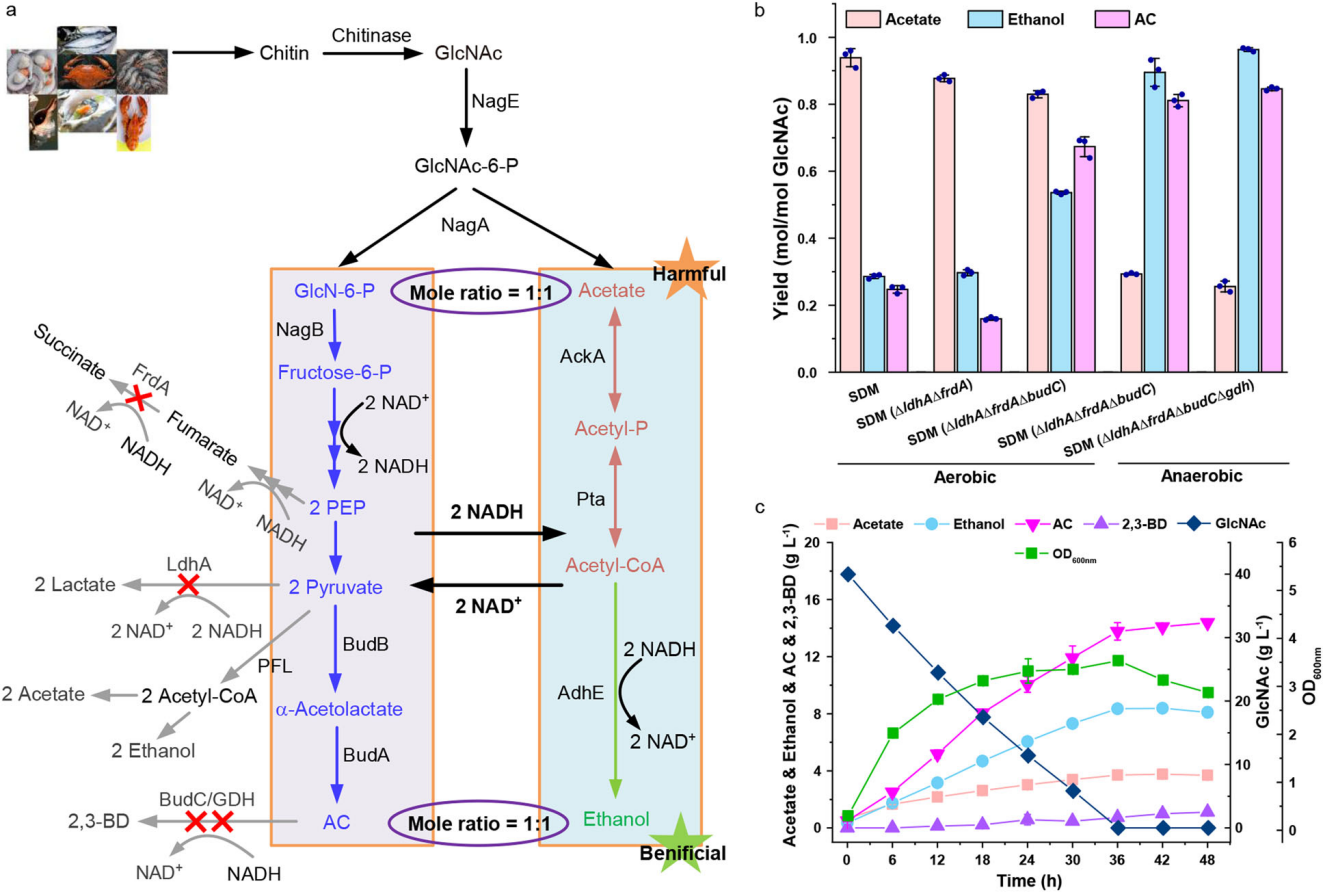
Production of AC and ethanol from chitin based on the acetate elimination mechanism in *E. cloacae* SDM. **a** Schematic diagram of chitin enzymolysis and GlcNAc metabolism in *E. cloacae* SDM (Δ*ldhA*Δ*frdA*Δ*budC*Δ*gdh*). NagE, N-acetylglucosamine specific PTS enzyme IIABC component; NagA, N-acetylglucosamine-6-phosphate deacetylase; NagB, glucosamine-6-phosphate deaminase; FrdA, fumarate reductase; LdhA, lactate dehydrogenase; PFL, pyruvate formate lyase; BudB, α-acetolactate synthase; BudA, α-acetolactate decarboxylase; BudC/GDH, 2,3-butanediol dehydrogenase; AckA, acetate kinase; Pta, phosphate acetyltransferase; AdhE, bifunctional acetaldehyde/ethanol dehydrogenase. **b** Yield of acetate, ethanol and AC produced by *E. cloacae* SDM and its derivatives under aerobic or anaerobic conditions using GlcNAc as the carbon source. **c** Batch fermentation using chitin hydrolysate as carbon source by *E. cloacae* SDM (Δ*ldhA*Δ*frdA*Δ*budC*Δ*gdh*) under anaerobic conditions. Data shown are means ± SD (*n* = 3 independent experiments).

Besides being a toxic metabolite produced by overflow metabolism, acetate is also an unavoidable component in many renewable resources. Chitin, the second most abundant polysaccharide in the world after cellulose^31^, is a natural polymer of GlcNAc, with a global annual turnover of about 100 billion tons. Catabolism of chitin inevitably involves the production of acetate^32^. Acetate detoxification and coordinated glucose metabolism are two major problems that must be solved to make chitin utilization technologically viable. To identify whether the acetate elimination mechanism could be applied in chitin bio-refinery, a combined catalytic and biotechnological approach for the production of AC (one of the 30 platform chemicals which are given the priority for development and utilization by the US Department of Energy)^33^ and ethanol from chitin was proposed (Fig. 5a).

During the consumption of GlcNAc, *E. cloacae* SDM continuously accumulated acetate with a yield of 0.92 mol per molar of GlcNAc (0.92 mol/mol GlcNAc) (Fig. 5b). To support NADH requiring acetate elimination, two NADH-consuming enzymes lactate dehydrogenase (LdhA) and fumarate reductase (FrdA) were blocked. *E. cloacae* SDM (Δ*ldhA*Δ*frdA*) can produce 2,3-BD with a lower acetate accumulation and a lower acetate yield (0.89 mol/mol GlcNAc). Besides *meso*-2,3-BD dehydrogenase encoded by *budC*, the (2*R*,3*R*)-2,3-BD dehydrogenase encoded by *gdh* can also consume NADH for AC reduction. Then, knockout of genes encoding these two 2,3-BD dehydrogenases and cultivation of mutant strains under anaerobic conditions were conducted to further supply NADH for acetate elimination and enable *E. cloacae* SDM producing AC and ethanol as its major products. Anaerobic cultivation of *E. cloacae* SDM (Δ*ldhA*Δ*frdA*Δ*budC*Δ*gdh*) eventually realized the co-production of AC (0.95 mol/mol GlcNAc) and ethanol (0.89 mol/mol GlcNAc) using GlcNAc as the substrate (Fig. 5b; Supplementary Fig. S5).

Chitin from the shrimp shell can be hydrolyzed by the chitinase of *Bacillus licheniformis*^34^. Then, anaerobic cultivation of *E. cloacae* SDM (Δ*ldhA*Δ*frdA*Δ*budC*Δ*gdh*) was conducted using chitin hydrolysate as the substrate. As shown in Fig. 5c, *E. cloacae* SDM (Δ*ldhA*Δ*frdA*Δ*budC*Δ*gdh*) produced 14.39 g L^−1^ AC and 8.10 g L^−1^ ethanol from chitin hydrolysate, with yields of 92% and 89% of the maximum theoretical yields, respectively.

## Discussion

**Fig. 6:**
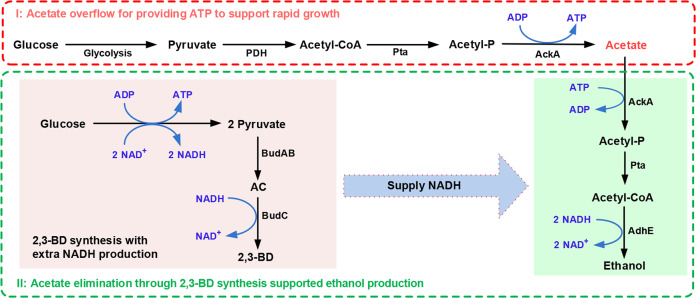
Mechanism of acetate-induced 2,3-BD biosynthesis supports ethanol production for acetate elimination. PDH, pyruvate dehydrogenase; Pta, phosphate acetyltransferase; AckA, acetate kinase; AdhE, bifunctional acetaldehyde/ethanol dehydrogenase; BudR, lysR family regulatory protein; BudA, α-acetolactate decarboxylase; BudB, α-acetolactate synthase; BudC, 2,3-butanediol dehydrogenase; Acetyl-P, acetyl phosphate.

2,3-BD is an important biochemical fuel with extensive industrial applications. Numerous strategies for microbial 2,3-BD production have been reported, whereas the metabolic function of 2,3-BD biosynthesis has not been well clarified. In this work, the physiological function of 2,3-BD biosynthesis was studied in *E. cloacae* SDM. A metabolic strategy in which 2,3-BD synthesis supports acetate elimination was proposed (Fig. 6). Acetate overflow leads to the initial accumulation of acetate in the early stages of *E. cloacae* SDM growth. Subsequently, acetate promotes the transcription of *budABC* and 2,3-BD synthesis supplies the reducing power required for AdhE-catalyzed ethanol production from acetyl-CoA. Compared with acetate catabolism through TCA and respiration, this strategy only requires three additional enzymes (BudA, BudB, and BudC) besides AdhE expressed during acetate overflow metabolism and can prevent the negative effects of acetate accumulation.

Acetate overflow is a bacterial allocation strategy to balance the proteomic demands of biomass synthesis and energy generation in order to achieve a high growth rate^2,35^. Acetate accumulation is one of the major problems restricting the industrial applications of some bacteria. However, simply blocking the acetate production (which would involve a decrease in energy generation)^36^ or enhancing acetate catabolism through respiration (with an associated increase in proteomic demand)^37^ cannot improve the fermentation performance of these bacteria. Besides being a toxic metabolite produced by overflow metabolism, acetate is also an unavoidable component in many renewable resources. Chitin, the second most abundant polysaccharide in the world after cellulose, is a natural polymer of GlcNAc. Catabolism of chitin involves the inevitable production of acetate. Based on the acetate elimination mechanism identified in this work, we solved the problem of acetate accumulation during GlcNAc utilization by efficiently channeling reducing power to ethanol production. Two important chemicals, AC and ethanol could be co-produced from chitin, an abundant renewable resource. In addition, acetate is an inevitable toxic compound in hemicellulose hydrolysate. By coupling NADH-consuming ethanol production from acetate with NADH-producing xylose catabolism, *S. cerevisiae* can be engineered to convert xylose and toxic acetate in hemicellulose hydrolysate into ethanol^4^. It should be noted that the production of 2,3-BD might also lead to wastage of the carbon source. Exogenously supplying reducing power by electrochemical methods for acetate elimination might be another viable alternative.

The concept of overflow has traditionally been associated with the redirection of carbon towards excreted organic acids by heterotrophic microbes. Recently, organic acid overflow was reported in a mutant cyanobacterium *Synechocystis* sp. PCC 6803, which is unable to synthesize the carbon storage compound glycogen. A further study has demonstrated that both glycogen synthesis and organic acid overflow contribute to energy balancing in cyanobacteria^38^. Glycogen synthesis may also be viewed as a unique overflow metabolism in autotrophic microbes, helping to balance energy homeostasis under changing environmental conditions. In this study, acetate overflow in fast-growing *E. cloacae* SDM was also observed (Fig. 1b). The accumulation of acetate would result in acidification of the medium, which would be harmful to the bacterial strain. Then, biosynthesis of non-toxic 2,3-BD was activated, and this process was shown to provide NADH for the transformation of harmful acetate to ethanol (Fig. 6). The results demonstrate an innovative overflow strategy whereby the production of a non-toxic chemical can be exploited to drive desirable metabolic reactions.

In summary, we have revealed that the coupling between 2,3-BD synthesis and ethanol production establishes a naturally existing strategy for acetate elimination. Physiological characterization of naturally occurring metabolic processes, such as 2,3-BD synthesis, might provide alternative methods for solving problems that result in challenges for the industrial applications of bacteria. This study, therefore, stresses the significance of research in classic metabolic pathways with unidentified functions.

## Materials and methods

### Materials

2,3-Butanediol (2,3-BD), AC, gluconic acid sodium salt, and sodium acetate-1-^13^C were purchased from Sigma (USA). N-acetylglucosamine (GlcNAc) was purchased from Aladdin (Shanghai, China). Restriction enzymes were purchased from TaKaRa Bio Inc. (China). Polymerase chain reaction (PCR) primers were provided by Sangon (Shanghai, China). FastPfu DNA polymerase and T4 DNA ligase were purchased from Transgen Biotech (China) and ThermoFisher (America), respectively. NAD^+^/NADH Quantification Colorimetric Kit was purchased from Biovision (K337-100, USA). EasyPure RNA Kit (used for RNA extraction) and TransStart Top Green qPCR SuperMix (used for qPCR) were purchased from Transgen (Beijing, China). HiScript II Q RT SuperMix for qPCR (+gDNA wiper, used for RNA reverse transcription) was purchased from Vazyme (R223-01, Nanjing, China). BacTiter-Glo™ Microbial Cell Viability Assay used for ATP detection was purchased from Promega (America). All other chemicals were of analytical grade and commercially available.

### Bacterial strains, plasmids, media, and culture conditions

All the strains and plasmids used in this study are listed in Supplementary Table S5. Lysogenic broth (LB) medium was used for the general culture of strains. M9 minimal medium^39^ supplemented with 5 g/L yeast extract and various carbon sources, including glucose, gluconic acid was used for the culture of *E. cloacae* SDM. Sodium hydrosulfite (5 g/L) and resazurin (1 mg/L) were added in M9 minimal medium for the anaerobic culture of *E. cloacae* SDM. Ampicillin and kanamycin were used at a concentration of 100 and 50 μg/mL, respectively. Experiments were carried out under aerobic conditions unless otherwise stated.

### Gene manipulation in bacterial strains

To construct the *E. cloacae* SDM (Δ*budABC*) mutant strain, the homologous arms upstream and downstream of *budABC* were amplified using primers Δ*budABC*-f1/Δ*budABC*-r2 and Δ*budABC*-f3/Δ*budABC*-r4 (Supplementary Table S6) from the genome of *E. cloacae* SDM. These two fragments were fused via PCR method using primers Δ*budABC*-f1 and Δ*budABC*-r4, which contained *EcoRI* and *BamHI* restriction enzyme sites, respectively. The infused fragment was linked with suicide plasmid pKR6K, which was also digested with *EcoRI* and *BamHI*. The resulting plasmid was designated pKR6K-Δ*budABC* and transferred into *E. coli* S17-1 for conjugation with *E. cloacae* SDM. The single-crossover mutants with the integration of pKR6K-Δ*budABC* into the chromosome were selected on an M9 agar plate supplemented with 20 g/L sodium citrate and 50 μg/mL kanamycin. The double-crossover mutants were selected on LB agar plates containing 15% (w/v) sucrose. All the constructed strains were validated by PCR and sequenced. The *adhE*, *budR*, *fnr*, *budC*, *pta*, *ackA,* and *acsA* mutants of *E. cloacae* SDM, *budABC,* and *adhE* mutants of *K. pneumoniae* ATCC 25955 were generated by using the same procedure. The primers used in this study were listed in Supplementary Table S6.

The knockout of *adhE* in *E. coli* K12 was conducted by the one-step inactivation method as described previously^39^. Briefly, *E. coli* K12-Δ*adhE*-f1/*E. coli* K12-Δ*adhE*-r2 (Supplementary Table S6) were used to directly amplify *adhE* mutant fragment from *adhE*::kan mutant strain purchased from The Coli Genetic Stock Center. The PCR products had ~330 bp up and down homologous arms, respectively, outside of *adhE* for homologous recombination and an FRT-flanked kanamycin resistance cassette for target gene replacement and mutant strain screening.

### Monitoring metabolic processes of strains

Unless specified, *E. cloacae* SDM, *K. pneumoniae* ATCC 25955, *E. coli* K12, and their derivatives were cultivated in M9 minimal medium supplemented with 5 g/L yeast extract and 40 g/L glucose as the sole carbon source at 37 °C and 180 rpm. Experiments were conducted in 300 mL shake flasks containing 50 mL of medium. Samples were withdrawn periodically to determine the cell density, concentrations of glucose, 2,3-BD, acetate, ethanol, and other by-products. To monitor the acetate elimination of various bacterial strains, about 10 mM ^13^C-labeled sodium acetate was added at the initial log-stage (at 2 h), and samples were withdrawn every 2 h to determine the concentrations of ^13^C-labeled acetate and ^13^C-labeled ethanol using gas chromatography–mass spectrometry (GC–MS, GCMS-QP2010 Plu, Shimadzu, Japan) and high-performance liquid chromatography (HPLC, Agilent 1100, America). Briefly, combined concentrations of labeled and unlabeled acetate were determined by HPLC. Concentrations of labeled acetate and unlabeled acetate were then detected by GC–MS. ^13^C-labeled acetate (^13^C acetate) was detected at *m*/*z* 61 and unlabeled acetate at *m*/*z* 60. The concentrations of ^13^C-labeled ethanol and unlabeled ethanol were analyzed with the same procedure.

### Quantitative real-time PCR

Total RNA was extracted from *E. cloacae* SDM or its derivatives using an EasyPure RNA Kit (Transgen, China). DNA contamination was eliminated by RNase-free DNase I (Vazyme, China), and the quality of extracted RNA was detected by 1.5% agarose gel electrophoresis and absorbance ratio at 260/280 nm. Then, cDNA was synthesized from total RNA by using HiScript II Q RT SuperMix (Vazyme, China).

The qPCR analysis was performed using TransStart Top Green qPCR SuperMix (Transgen, China) on a LightCycler 480 system (Roche). For absolute quantification of the copy numbers, standard curves were constructed for *adhE* and *budA* by serial dilutions of the recombinant plasmids harboring the same amplicons. Each reaction was performed in triplicate. Controls with no template or total RNA were included for each reaction on the same plate. The primers used for qPCR analysis are listed in Supplementary Table S6.

### Construction of standard curves for qPCR

Partial *budA* gene sequences were amplified by PCR using primers *budA*-qPCR (SDM-f)/*budA*-qPCR (SDM-r) (Supplementary Table S6) and then cloned into pEASY-Blunt Simple Cloning Vector (Transgen, China) to form pEASY-Blunt-*budA*. This plasmid was extracted by using Plasmid Miniprep Kit (Biomiga, USA) and quantified by using NanoDrop ND-1000 (Thermo Scientific, USA). The copy number of the recombinant plasmids was calculated based on the molecular weight^40^. The standard curve for qPCR was constructed with serial 10-fold dilutions of a recombinant plasmid, ranging from 1 × 10^5^ to 1 × 10^10^ copies per μL. For each set, determined threshold cycle (C_T_) values were plotted against the logarithm of their known initial copy number (per μL) using Origin software 9.0 (OriginLab, USA). PCR amplification efficiency (*E*) was calculated according to the equation: *E* = 10^−1/slope^–1 as described previously^41^. The standard curve for qPCR of *adhE* was generated by using the same procedure. All of the reactions and controls with no template were run in triplicate.

### Sample preparation for qPCR

*E. cloacae* SDM and its derivatives were grown overnight in LB medium and then inoculated into M9 minimal medium supplemented with 5 g/L yeast extract and 40 g/L glucose as the sole carbon source. Cells of these strains were harvested every two hours for the quantification of mRNA. Cells from LB medium were termed as the sample at 0 h. To identify the effect of acetate on transcription of *budA* and *adhE*, the procedure was the same as above except that 40 mM sodium acetate was added into M9 minimal medium at the beginning.

### β-Galactosidase assay

The promoter region of the *adhE* gene (P_*adhE*_) was amplified from *E. cloacae* SDM genomic DNA using primers P_*adhE*_ (SDM-f)/P_*adhE*_ (SDM-r) (Supplementary Table S6). Then, the purified PCR product was ligated with pME6522 to generate pME6522-P_*adhE*_. In the same way, the promoter region of *budA* gene (P_*budA*_) was cloned into pME6522 to generate pME6522-P_*budA*_. pME6522-P_*budA*_ and pME6522-P_*adhE*_ were transferred into *E. cloacae* SDM and its derivatives by electroporation.

*E. cloacae* SDM and its derivatives harboring pME6522-P_*budA*_ or pME6522-P_*adhE*_ were grown in M9 minimal medium supplemented with 5 g/L yeast extract and 40 g/L glucose as sole carbon source and harvested at the mid-log stage by centrifugation at 8000 rpm for 5 min. The β-galactosidase activity was then determined using *o*-nitrophenyl-β-d-galactopyranoside as the substrate.

### Detection of intracellular NADH/NAD^+^ level

*E. cloacae* SDM and its derivatives were cultured in an M9 minimal medium supplemented with 5 g/L yeast extract and 40 g/L glucose as the sole carbon source to mid-log phase. Then, *E. cloacae* cells were collected by centrifugation at 13,000 rpm for 1 min. The intracellular concentrations of NADH and NAD^+^ were determined using the NAD^+^/NADH Quantification Colorimetric Kit (BioVision, K227-100, America) according to the manufacturers’ instructions.

### Detection of ATP level

*E. cloacae* SDM and *E. cloacae* SDM (Δ*budABC*) were grown in an M9 minimal medium supplemented with 5 g/L yeast extract and 40 g/L glucose and harvested at mid-log phase. The ATP levels were determined using the BacTiter-Glo™ Microbial Cell Viability Assay (Promega, America) according to the manufacturers’ instructions.

### Determination of survival rate

*E. cloacae* SDM and *E. cloacae* SDM (Δ*budABC*) were grown in M9 minimal medium supplemented with 5 g/L yeast extract and 40 g/L glucose. Cells were harvested at 6, 30, 54, 78, 102 h with centrifugation at 8000 rpm for 2 min. SYTO 9 and propidium iodide included in a LIVE/DEAD BacLight Bacterial Viability Kits (Invitrogen, L7012) were used for the determination of survival rate at different times. The number of live/dead cells was counted by a quantitative imaging analysis flow cytometer (ImageStream^x^ Mark II) with an exciting light at 480 nm.

### Expression and purification of chitinase

The gene encoding chitinase was amplified from the genome of *B. licheniformis* DSM13 by PCR using primers *chi*-f/*chi*-r (Supplementary Table S6). The PCR product and plasmid pFLAG-CTS were digested by HindIII and XhoI and then linked by T4 DNA ligase to construct plasmid pFLAG-CTS-*chi*. Then, the plasmid pFLAG-CTS-*chi* was transformed into *E. coli* Top10 for chitinase expression.

Purification of chitinase was conducted as described by a group of Songsiriritthigul with slight modification^34^. In brief, the recombinant strain *E. coli* Top10-pFLAG-CTS-*chi* was cultured in LB medium containing 100 µg/mL ampicillin at 37 °C to an optical density at 600 nm of 1.5, then 1 mM IPTG was added to induce protein expression under 25 °C for 18 h. After that, cells were harvested by centrifugation at 6000 rpm for 10 min and washed twice by 0.85% saline solution. The freshly prepared cell pellet was resuspended in lysis buffer (OD_600_ reached approximately 30), then lysed on ice using a high-pressure crusher. Cell debris and intact cells were removed by centrifugation at 12,000 rpm and 4 °C for 30 min. The resultant supernatants were immediately applied to a HisTrap HP column (5 mL) equilibrated with binding buffer (20 mM sodium phosphate and 500 mM sodium chloride, pH 7.4) and eluted with a gradient ratio of wash buffer (20 mM sodium phosphate, 500 mM imidazole, and 500 mM sodium chloride, pH 7.4) to obtain purified chitinase (Supplementary Fig. S6).

### Co-production of AC and ethanol from GlcNAc or chitin hydrolysate

Chitin hydrolysate was prepared from colloidal chitin according to the method of Songsiriritthigul et al. ^34^ with some modification. Briefly, 50 g of chitin powder (Aladdin, China) was slowly added into 500 mL concentrated HCl with vigorous stirring for 3−4 h and then incubated overnight. The mixture was filtered through a cheesecloth, dropped slowly into 600 mL of ice-cold ethanol (50%) with rapid stirring on ice. The colloidal chitin was collected by centrifugation at 8000 rpm for 10 min and washed several times with tap water until the pH approached neutral. Then, recombinant chitinase of *B. licheniformis* was added into sterilized colloidal chitin (200 g/L) at a concentration of 0.5 U/mL and the mixture was incubated at 50 °C and 180 rpm for 48 h.

For the co-production of AC and ethanol, *E. cloacae* SDM and its derivatives were cultured in M9 minimal medium supplemented with 5 g/L yeast extract and 40 g/L GlcNAc under aerobic or anaerobic condition. Alternatively, the chitin hydrolysate was fed into the M9 minimal medium to make the GlcNAc concentration at about 40 g/L. Anaerobic culture was conducted with 50 mL anaerobic bottles containing 40 mL of medium in the anaerobic incubator.

### Analytical methods

Cell density was measured using a spectrophotometer (Lengguang-721, China) at a wavelength of 600 nm. The concentration of glucose was measured enzymatically by a bio-analyzer (SBA-40D, Shandong Academy of Sciences, China) after diluting to an appropriate concentration. The pH of the culture was measured by an Orion Star A211 pH meter. The concentration of 2,3-BD and AC were determined by GC (GC2014C, Shimadzu, Japan) using a capillary GC column as described previously^42^. Other metabolites e.g. acetate, ethanol, lactate, and succinate were measured using high-performance liquid chromatography (HPLC, Agilent 1100, America) as described^39^.

### Statistical analysis

Software for initial data processing was Microsoft Excel 2013, and subsequent analysis was carried out using Origin (OriginLab), GraphPad Prism 5 (GraphPad). Venn diagrams of the distribution of AdhE and BudA were drawn by the webtool in Bioinformatics & Systems Biology. Analogous enzymes of AdhE and BudA were gained by NCBI Blast online. Detailed data analysis is described in the text.

## Supplementary information

Fig. S1

Fig. S2

Fig. S3

Fig. S4

Fig. S5

Fig. S6

Table S1

Table S2

Table S3

Table S4

Table S5

Table S6

## Data Availability

All data needed to support the conclusions in the paper are present in the paper and/or the Supplementary Materials. Additional data related to this paper may be requested from the authors.
