# Supplementary material for: 2,3-Butanediol synthesis from glucose supplies NADH for elimination of toxic acetate produced during overflow metabolism
Source: Cell Discov. 2021 Jun 8;7:43. doi: 10.1038/s41421-021-00273-2 (PMC8187413; doi:10.1038/s41421-021-00273-2)
Supplement: Supplementary file 1 — Fig. S1 [file 41421_2021_273_MOESM1_ESM.pdf]

Supplementary Materials

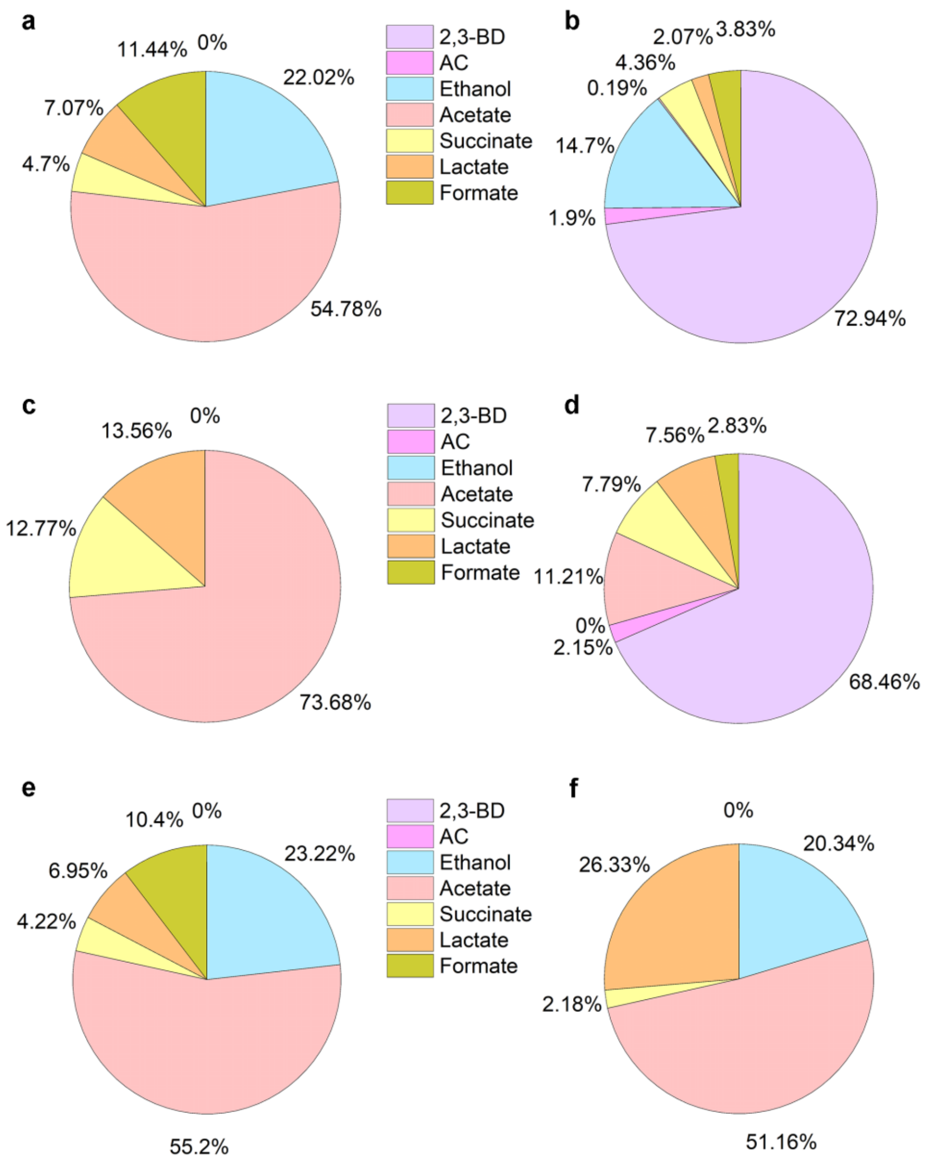

Supplementary Fig. S1 AdhE and BudABC participate in acetate elimination of *E. cloacae* SDM. **a**, **b** Metabolic flux redistribution of *E. cloacae* SDM at 2 h and 6 h, respectively. **c**, **d** Metabolic fluxes of *E. cloacae* SDM (Δ*adhE*) at 2 h and 6 h, respectively. **e**, **f** Metabolic fluxes of *E. cloacae* SDM (Δ*budABC*) at 2 h and 6 h, respectively. Strains were cultured in M9 minimal medium supplemented with 5 g L^{-1} yeast extract and 40 g L^{-1} glucose at 37 ℃ and 180 rpm. Metabolic fluxes of strains at 2 h and 6 h were calculated based on the determination of major metabolite concentrations. Experiments were carried out under aerobic conditions. The data came from three biological parallel experiments.
