# Supplementary material for: 2,3-Butanediol synthesis from glucose supplies NADH for elimination of toxic acetate produced during overflow metabolism
Source: Cell Discov. 2021 Jun 8;7:43. doi: 10.1038/s41421-021-00273-2 (PMC8187413; doi:10.1038/s41421-021-00273-2)
Supplement: Supplementary file 2 — Fig. S2 [file 41421_2021_273_MOESM2_ESM.pdf]

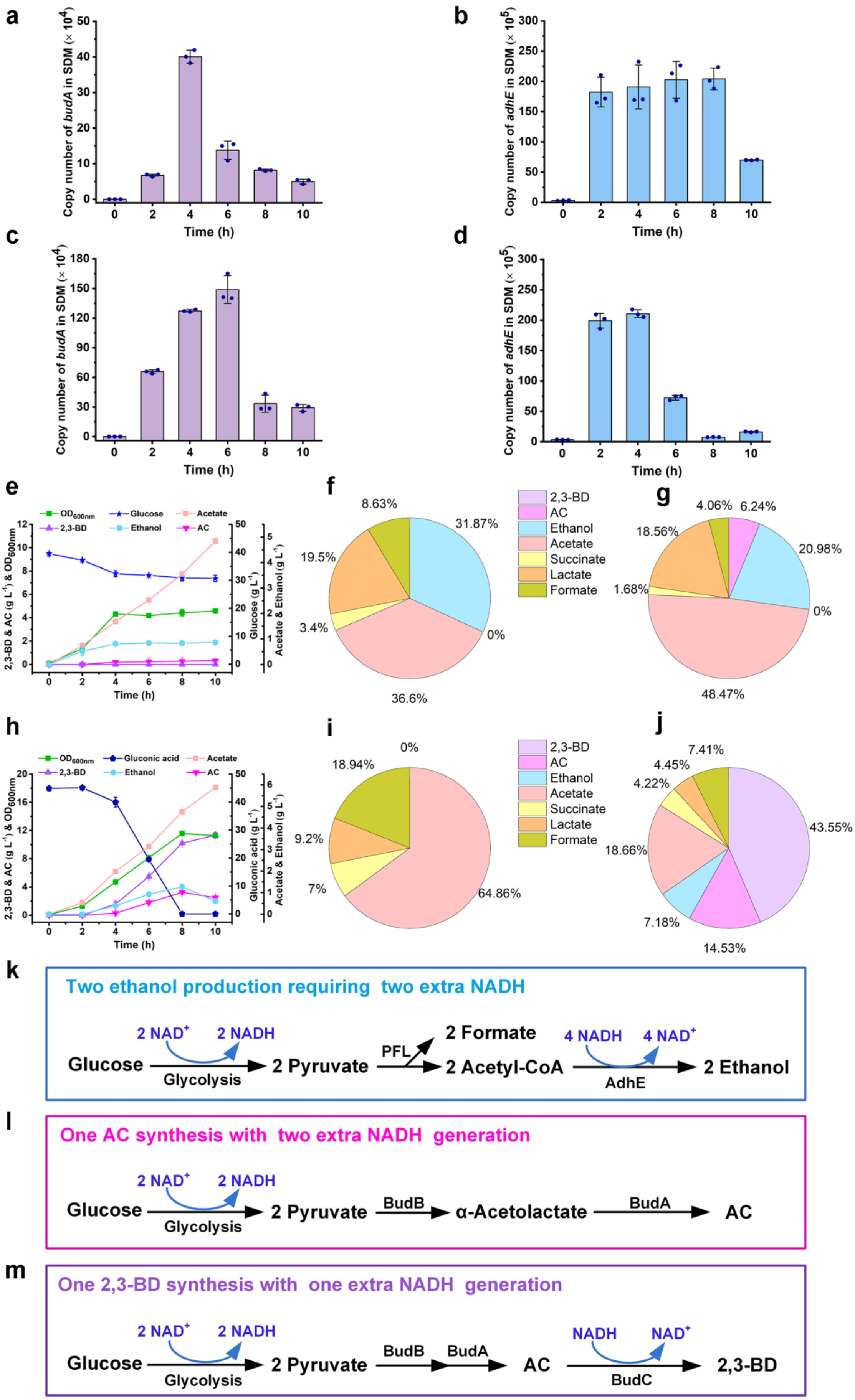
a
Copy number of budA in SDM (× 10^4)
Time (h)
b
Copy number of adhE in SDM (× 10^5)
Time (h)
c
Copy number of budA in SDM (× 10^4)
Time (h)
d
Copy number of adhE in SDM (× 10^5)
Time (h)
e
OD600nm
Glucose
Acetate
2,3-BD
Ethanol
AC
2,3-BD & AC (g L^-1) & OD600nm
Glucose (g L^-1)
Acetate & Ethanol (g L^-1)
Time (h)
f
8.63%
31.87%
19.5%
3.4%
0%
36.6%
2,3-BD
AC
Ethanol
Acetate
Succinate
Lactate
Formate
g
4.06%
6.24%
18.56%
20.98%
1.68%
0%
48.47%
h
OD600nm
Gluconic acid
Acetate
2,3-BD
Ethanol
AC
2,3-BD & AC (g L^-1) & OD600nm
Gluconic acid (g L^-1)
Acetate & Ethanol (g L^-1)
Time (h)
i
0%
18.94%
9.2%
7%
64.86%
2,3-BD
AC
Ethanol
Acetate
Succinate
Lactate
Formate
j
7.41%
4.45%
4.22%
43.55%
18.66%
7.18%
14.53%
k
Two ethanol production requiring two extra NADH
2 NAD+
2 NADH
Glucose
Glycolysis
2 Pyruvate
PFL
2 Formate
2 Acetyl-CoA
4 NADH
4 NAD+
AdhE
2 Ethanol
l
One AC synthesis with two extra NADH generation
2 NAD+
2 NADH
Glucose
Glycolysis
2 Pyruvate
BudB
α-Acetolactate
BudA
AC
m
One 2,3-BD synthesis with one extra NADH generation
2 NAD+
2 NADH
Glucose
Glycolysis
2 Pyruvate
BudB
BudA
AC
NADH
NAD+
BudC
2,3-BD

Supplementary Fig. S2 Mechanism of 2,3-BD biosynthesis supported acetate elimination. **a**, **b** The copy number of *budA* and *adhE* in *E. cloacae* SDM during the growth in glucose. **c**, **d** The copy number of *budA* and *adhE* in *E. cloacae* SDM during the growth in glucose supplemented with 40 mM sodium acetate. **e** Growth of *E. cloacae* SDM (Δ*budR*) in M9 minimal medium supplemented with 5 g L^{-1} yeast extract and 40 g L^{-1} glucose, concentrations of glucose, AC, 2,3-BD, acetate and ethanol were assayed. **f**, **g** Metabolic fluxes of *E. cloacae* SDM (Δ*budR*) at 2 h and 6 h, respectively. The data came from three biological parallel experiments. **h** Growth of *E. cloacae* SDM in M9 minimal medium supplemented with 5 g L^{-1} yeast extract and 40 g L^{-1} gluconic acid, concentrations of gluconic acid, AC, 2,3-BD, acetate and ethanol were assayed. **i**, **j** Metabolic fluxes of *E. cloacae* SDM grown in conditions as stated in **h** at 2 h and 6 h, respectively. The data came from three biological parallel experiments. Metabolic fluxes of strains at 2 h and 6 h were calculated based on the determination of major metabolite concentrations. **k**, **l**, **m** Redox balance analysis under anaebolic condition of the metabolic pathways of ethanol, AC and 2,3-BD production, respectively. PFL, pyruvate formate lyase; AdhE, bifunctional acetaldehyde/ethanol dehydrogenase; BudB, α-acetolactate synthase; BudA, α-acetolactate decarboxylase; BudC, 2,3-butanediol dehydrogenase. Data shown are mean ± s.d. (n = 3 independent experiments). Experiments were carried out under aerobic conditions.
