# Supplementary material for: 2,3-Butanediol synthesis from glucose supplies NADH for elimination of toxic acetate produced during overflow metabolism
Source: Cell Discov. 2021 Jun 8;7:43. doi: 10.1038/s41421-021-00273-2 (PMC8187413; doi:10.1038/s41421-021-00273-2)
Supplement: Supplementary file 3 — Fig. S3 [file 41421_2021_273_MOESM3_ESM.pdf]

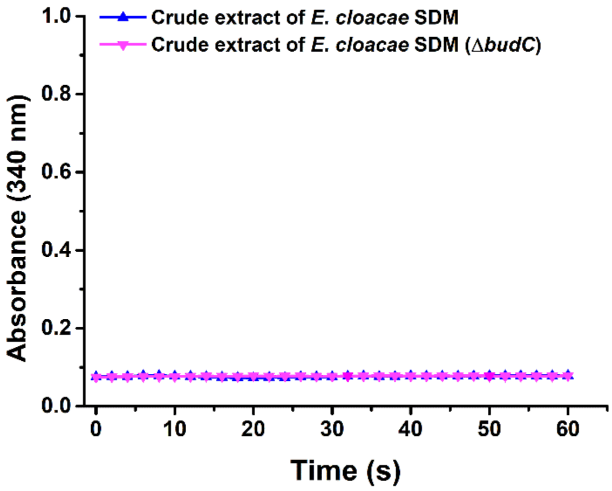

Supplementary Fig. S3 Assay of formate dehydrogenase (FDH) activity in *E. cloacae* SDM and *E. cloacae* SDM (Δ*budC*). The assay mixture contained 67 mM phosphate buffer (pH 7.4), 5 mM sodium formate and 1 mM NAD^+. After adding the crude extracts of *E. cloacae* SDM or *E. cloacae* SDM (Δ*budC*), the variation of NADH absorbance at 340 nm were recorded with a UV/visible spectrophotometer (Ultrospec 2100 pro, Amersham Biosciences, USA).
