# Supplementary material for: 2,3-Butanediol synthesis from glucose supplies NADH for elimination of toxic acetate produced during overflow metabolism
Source: Cell Discov. 2021 Jun 8;7:43. doi: 10.1038/s41421-021-00273-2 (PMC8187413; doi:10.1038/s41421-021-00273-2)
Supplement: Supplementary file 4 — Fig. S4 [file 41421_2021_273_MOESM4_ESM.pdf]

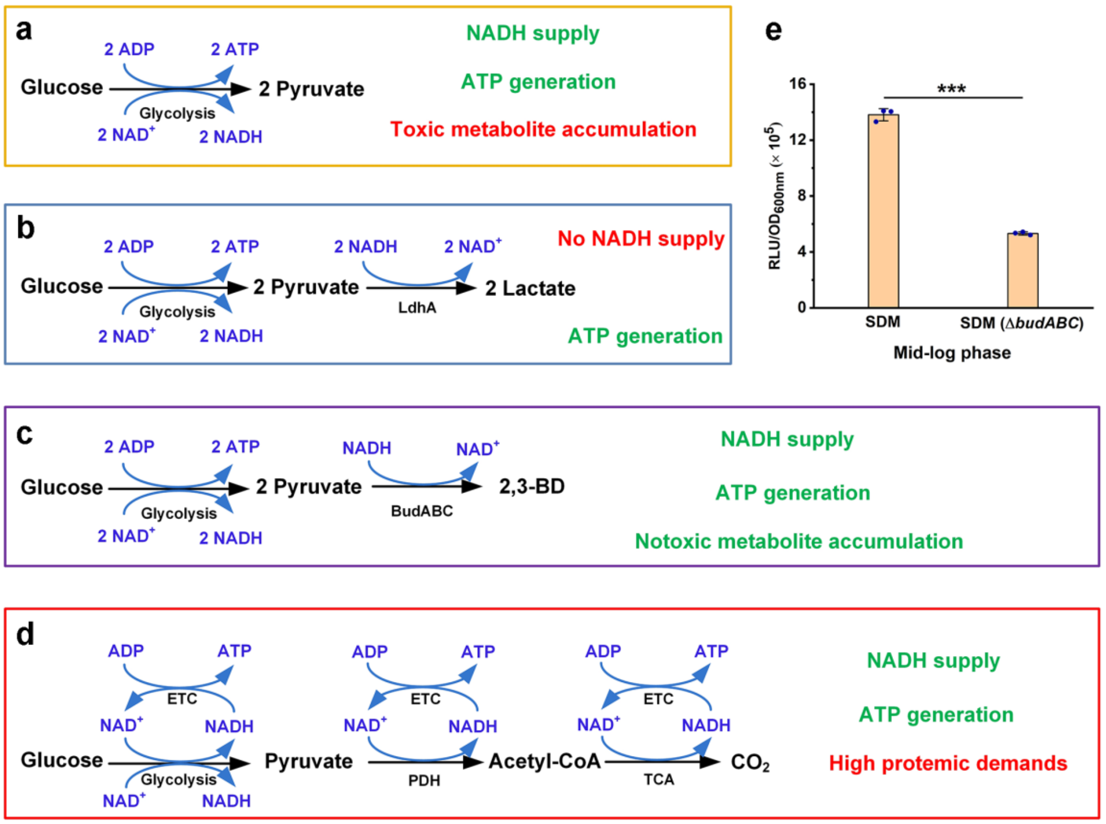

Supplementary Fig. S4 2,3-BD synthesis pathway is a better choice for strains among different metabolic processes. a Metabolic process of pyruvate synthesis. **b** Metabolic process of lactate production. **c** Metabolic process of 2,3-BD generation. **d** Metabolic process of oxidative phosphorylation. LdhA, lactate dehydrogenase; BudA, α-acetolactate decarboxylase; BudB, α-acetolactate synthase; BudC, 2,3-butanediol dehydrogenase; PDH, pyruvate dehydrogenase; TCA, tricarboxylic acid cycle; ETC, electron transport chain. **e** Mutation of 2,3-BD synthesis pathway decreased ATP level in *E. cloacae* SDM (two-tailed t test, ***, $P < 0.001$). *E. cloacae* SDM and *E. cloacae* SDM (Δ*budABC*) were cultured in M9 minimal medium supplemented with 5 g L^{-1} yeast extract and 40 g L^{-1} glucose at 37 ℃ and 180 rpm and harvested at mid-log phase for ATP detection. Experiments were carried out under aerobic conditions. Data shown are mean ± s.d. (n = 3 independent experiments).
