# Supplementary material for: 2,3-Butanediol synthesis from glucose supplies NADH for elimination of toxic acetate produced during overflow metabolism
Source: Cell Discov. 2021 Jun 8;7:43. doi: 10.1038/s41421-021-00273-2 (PMC8187413; doi:10.1038/s41421-021-00273-2)
Supplement: Supplementary file 5 — Fig. S5 [file 41421_2021_273_MOESM5_ESM.pdf]

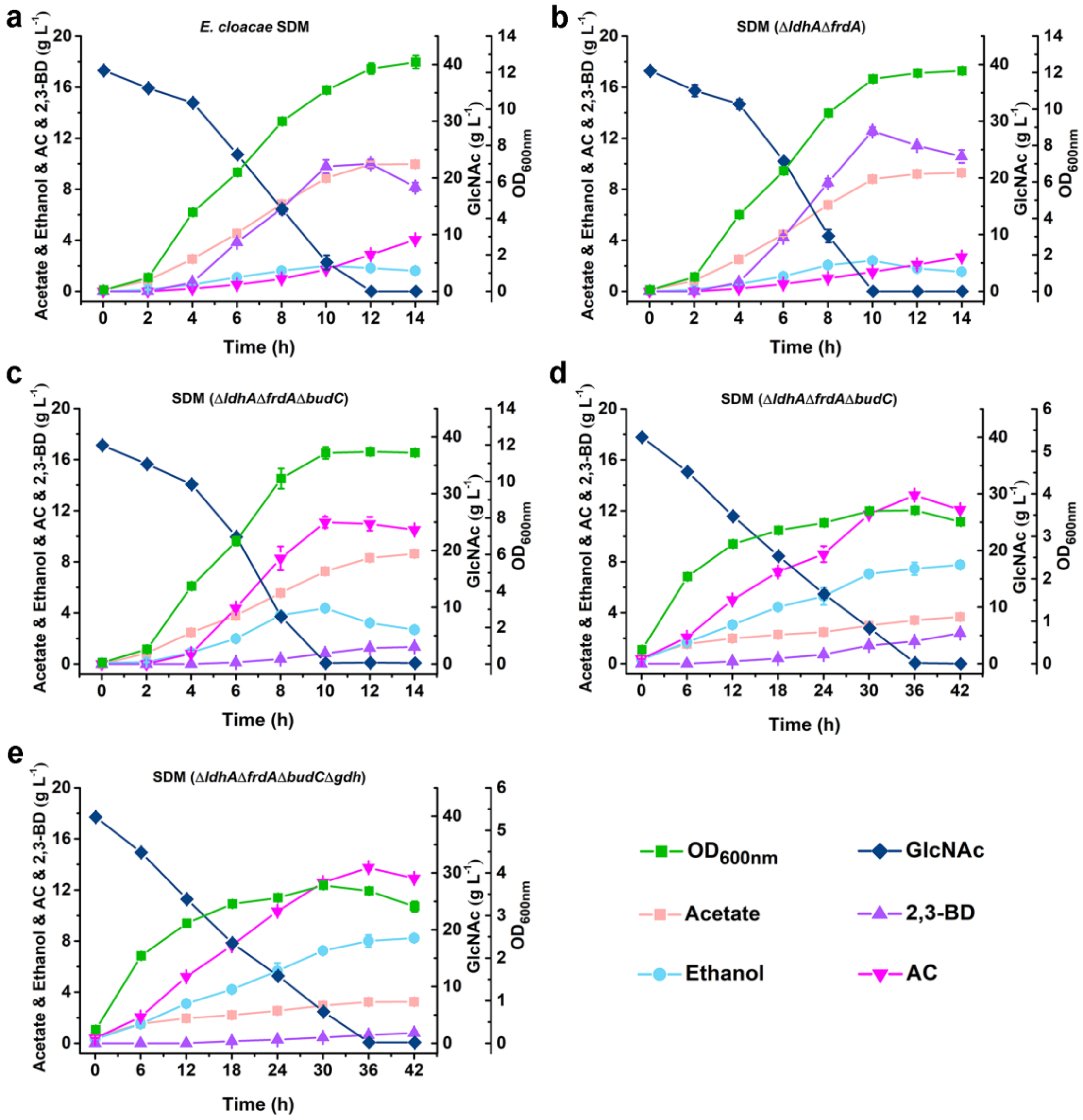

Supplementary Fig. S5 Batch fermentation using N-acetylglucosamine (GlcNAc) as carbon source by *E. cloacae* SDM and its derivatives. a *E. cloacae* SDM under aerobic condition. **b** *E. cloacae* SDM (Δ*ldhA*Δ*frdA*) under aerobic condition. **c** *E. cloacae* SDM (Δ*ldhA*Δ*frdA*Δ*budC*) under aerobic condition. **d** *E. cloacae* SDM (Δ*ldhA*Δ*frdA*Δ*budC*) under anaerobic condition. **e** *E. cloacae* SDM (Δ*ldhA*Δ*frdA*Δ*budC*Δ*gdh*) under anaerobic condition. Data shown are mean ± s.d. (n = 3 independent experiments).
