# Supplementary material for: 2,3-Butanediol synthesis from glucose supplies NADH for elimination of toxic acetate produced during overflow metabolism
Source: Cell Discov. 2021 Jun 8;7:43. doi: 10.1038/s41421-021-00273-2 (PMC8187413; doi:10.1038/s41421-021-00273-2)
Supplement: Supplementary file 6 — Fig. S6 [file 41421_2021_273_MOESM6_ESM.pdf]

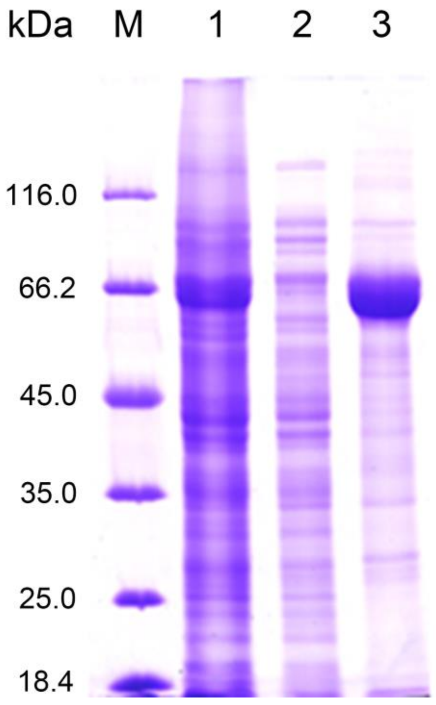

Supplementary Fig. S6 SDS-PAGE of expression and purification steps of chitinase. Lane M, molecular weight markers; lane 1, crude extract of *E. coli* Top10 harboring pFLAG-CTS-*chi*; lane 2, the unbound protein of the HisTrap HP column; lane 3, purified chitinase using a HisTrap column.
