# Supplementary material for: 2,3-Butanediol synthesis from glucose supplies NADH for elimination of toxic acetate produced during overflow metabolism
Source: Cell Discov. 2021 Jun 8;7:43. doi: 10.1038/s41421-021-00273-2 (PMC8187413; doi:10.1038/s41421-021-00273-2)
Supplement: Supplementary file 7 — Table S1 [file 41421_2021_273_MOESM7_ESM.pdf]

Supplementary Table S1 Concentrations (mM) of ^{13}C acetate and ^{13}C ethanol in *E. cloacae* SDM and its derivatives at 2 h and 6 h as well as the yield (mol/mol) of ^{13}C ethanol from ^{13}C acetate.

Strain	SDM	SDM (Δ*budABC*)	SDM (Δ*adhE*)
^{13}C Acetate (2 h)	8.04 ± 0.25	8.49 ± 0.52	8.01 ± 0.18
^{13}C Ethanol (2 h)	0.00 ± 0.00	0.00 ± 0.00	0.00 ± 0.00
^{13}C Acetate (6 h)	0.29 ± 0.13	6.58 ± 0.20	6.97 ± 0.15
^{13}C Ethanol (6 h)	6.22 ± 0.49	0.56 ± 0.13	0.00 ± 0.00
Yield	0.80 ± 0.03	0.29 ± 0.02	0.00 ± 0.00
