# Supplementary material for: 2,3-Butanediol synthesis from glucose supplies NADH for elimination of toxic acetate produced during overflow metabolism
Source: Cell Discov. 2021 Jun 8;7:43. doi: 10.1038/s41421-021-00273-2 (PMC8187413; doi:10.1038/s41421-021-00273-2)
Supplement: Supplementary file 8 — Table S2 [file 41421_2021_273_MOESM8_ESM.pdf]

Supplementary Table S2 Occurrence of genes encoding BudA and AdhE[a].

Organism	BudA	AdhE
**Gammaproteobacteria**	**161**	**508**
Enterobacter cloacae	+	+
Klebsiella pneumoniae	+	+
Vibrio anguillarum	+	+
E. coli	−	+
Erwinia rhapontici	+	−
Pantoea alhagi	+	+
**Betaproteobacteria**	**13**	**44**
Candidatus paraburk	+	−
Andreprevotia chitinilytica	−	+
**Actinobacteria**	**12**	**23**
Mycobacterium marinum	+	−
Collinsella aerofaciens	−	+
**Firmicutes**	**1**	**602**
Clostridium merdae	−	+
**Alphaproteobacteria**	**1**	**31**
**Deltaproteobacteria**	**0**	**7**
**Cyanobacteria**	**0**	**71**
**Spirochaetes**	**0**	**27**
**Acidobacteria**	**0**	**3**
**Fusobacteria**	**0**	**33**
**Bacteroidetes**	**0**	**12**
**Planctomycetes**	**0**	**3**
**Chloroflexi**	**0**	**3**
**Thermotogae**	**0**	**3**
**Tenericutes**	**0**	**9**
**Synergistetes**	**0**	**5**
**Elusimicrobia**	**0**	**1**
**Fibrobacteres**	**0**	**2**
**Kiritimatiellaeota**	**0**	**1**
**Lentisphaerae**	**0**	**1**
**Total**	**188**	**1389**

[a]Number of species
