# Supplementary material for: 2,3-Butanediol synthesis from glucose supplies NADH for elimination of toxic acetate produced during overflow metabolism
Source: Cell Discov. 2021 Jun 8;7:43. doi: 10.1038/s41421-021-00273-2 (PMC8187413; doi:10.1038/s41421-021-00273-2)
Supplement: Supplementary file 9 — Table S3 [file 41421_2021_273_MOESM9_ESM.pdf]

Supplementary Table S3 Fluctuation of extracellular pH during the culture of *E. cloacae* SDM and *E. cloacae* SDM (Δ*budABC*).

Time (h)	0	2	4	6	8
SDM	7.21 ±0.02	6.87 ±0.01	6.03 ±0.01	5.75 ±0.01	5.72 ±0.02
SDM (Δ*budABC*)	7.21 ±0.04	6.99 ±0.01	5.10 ±0.02	4.72 ±0.01	4.72 ±0.01
