# Supplementary material for: 2,3-Butanediol synthesis from glucose supplies NADH for elimination of toxic acetate produced during overflow metabolism
Source: Cell Discov. 2021 Jun 8;7:43. doi: 10.1038/s41421-021-00273-2 (PMC8187413; doi:10.1038/s41421-021-00273-2)
Supplement: Supplementary file 10 — Table S4 [file 41421_2021_273_MOESM10_ESM.pdf]

Supplementary Table S4 Survival rate of *E. cloacae* SDM and *E. cloacae* SDM (Δ*budABC*)[a].

Time (h)	6	30	54	78	102
SDM	99.37 ±0.40	98.83 ±0.38	96.03 ±3.34	97.97 ±1.21	95.97 ±2.11
SDM (Δ*budABC*)	97.70 ±2.25	95.83 ±1.31	0.19 ±0.07	0.15 ±0.03	0.12 ±0.05

[a]Survival rate (%) was obtained through dividing the number of cells alive by the total number of cells.
